# Leveraging Saliva for Insights into Head and Neck Cancer

**DOI:** 10.3390/ijms252413514

**Published:** 2024-12-17

**Authors:** Saad Rashid, Prashant Puttagunta, Saagar Pamulapati, Jianqiang Yang, Suneha Pocha, Nabil F. Saba, Yong Teng

**Affiliations:** 1 Internal Medicine Program, Mercyhealth Graduate Medical Education Consortium, Rockford, IL 61114, USA; sarashid@mhemail.org (S.R.); spocha@mhemail.org (S.P.); 2 Medical Education, University of Michigan Medical School, Ann Arbor, MI 48105, USA; puttagun@med.umich.edu; 3 Hematology-Oncology, Advocate Lutheran General Hospital, Park Ridge, IL 60068, USA; spamul2@uic.edu; 4 Department of Hematology and Medical Oncology, Emory University, Atlanta, GA 30322, USA; jianqiang.yang@emory.edu (J.Y.);; 5 Winship Cancer Institute, Emory University, Atlanta, GA 30322, USA; 6 Wallace H. Coulter Department of Biomedical Engineering, Georgia Institute of Technology, Emory University, Atlanta, GA 30322, USA

**Keywords:** saliva, biomarkers, head and neck cancer, biopsy, diagnosis

## Abstract

Head and neck cancer (HNC) represents a heterogeneous group of malignancies with increasing global incidence and notable mortality. Early detection is essential for improving survival rates and minimizing recurrence; however, existing diagnostic methods are often invasive and complex. There is a need for noninvasive and more effective approaches for early detection and real-time monitoring of HNC. Saliva contains various biomolecules that may serve as indicators of HNC. As a result, saliva-based biomarkers have emerged as a transformative approach in the diagnosis and treatment of HNC due to their ease of collection, non-invasiveness, and potential to provide details about biomolecular changes associated with cancer progression. This narrative review synthesizes the current literature on the potential of saliva as a noninvasive diagnostic tool for HNC. It highlights various biomarkers found in saliva, including cell-free DNA, RNA, proteins, and metabolites, and explores emerging technologies in saliva detection that could transform the future of HNC management. Continued research efforts and larger-scale validation studies are essential to fully realize the potential of saliva-based biopsy and help pinpoint notable biomarkers to improve patient outcomes and reduce mortality associated with HNC worldwide.

## 1. Introduction

Head and neck cancer (HNC) is the seventh most common cancer worldwide, posing high mortality and morbidity for patients [1]. Predisposing factors include alcohol use, tobacco use, human papillomavirus (HPV) infection, wood dust exposure, poor oral hygiene, and genetic predisposition [2]. It is self-evident that earlier diagnosis of cancer would be expected to result in earlier stage disease, improved prognosis, with possibly better tolerated intervention. Similarly, earlier detection of recurrence would be expected to offer an improved likelihood of effective management.

A majority of diagnoses are made at advanced stages of disease. Currently, there is not a standardized or widely recommended technology or method for early screening applicable to the general population in HNC. An endoscope or radiologic imaging can be performed for high-risk populations but is not suitable for the general population and also cannot provide the basis for early intervention at the molecular level of tumor development. Hence, efforts have been made to identify potential biomarkers or specific techniques that could be effective for screening in HNC. Given the anatomical proximity and tissue specificity, saliva is clearly a more suitable medium for developing early diagnostic methods. It has also emerged as a research hotspot in cancer screening due to its numerous advantages. Firstly, collecting saliva is non-invasive and painless compared to blood draws or tissue biopsies. This can increase patient compliance and comfort, making it easier to conduct repeated tests. Secondly, saliva samples can be collected easily without the need for specialized medical personnel, making it more accessible for people and feasible for large-scale screenings or remote locations. Finally, saliva screening could potentially detect HNC at an earlier stage when treatment outcomes are generally better, due to its ability to reveal genetic and molecular changes associated with cancer [3,4].

**Figure 1:**
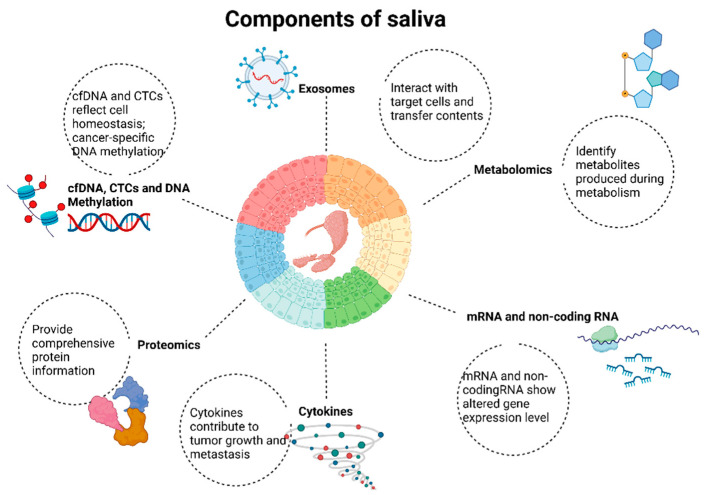
The various components present in saliva and their potential applications. At the center are the salivary glands, surrounded by different biomolecules derived from saliva: (1) cfDNA in saliva reflects cellular homeostasis and can exhibit cancer-specific DNA methylation patterns. (2) Exosomes in saliva interact with target cells and transfer their contents, playing a role in cell-to-cell communication and serving as potential biomarkers for diagnostics. (3) Metabolites in saliva produced during metabolism assist in understanding metabolic changes. (4) mRNA and miRNA in saliva show altered gene expression levels, indicating the presence of diseases and monitoring physiological states. (5) Cytokines in saliva contribute to tumor growth and metastasis, serving as indicators of inflammatory responses and cancer progression. (6) Proteins in saliva provide comprehensive information on the protein content in saliva, offering insights into mechanisms and identifying potential biomarkers. Components isolated from saliva and their biological functions: cfDNA and DNA methylation.

In this paper, we conduct a comprehensive review of salivary biomarkers for the detection, prognosis, and treatment monitoring of HNC, along with their underlying mechanisms. We discuss recent technological advances that have enabled in-depth characterization of the complex array of biomolecules in saliva and their role in detecting HNC, which includes transcriptomic, genomic, proteomic, and metabolomic changes (Figure 1) present in circulating analytes of saliva that can provide real-time monitoring of HNC patients [5].

## 2. Components Isolated from Saliva and Their Biological Functions

### 2.1. Cell-Free DNA (cfDNA)/Circulating Tumor DNA (ctDNA) and DNA Methylation

Cancer cfDNA, also known as ctDNA, is fragmented DNA found in various bodily fluids, including plasma, serum, and saliva. Physiologically, cfDNA is thought to be derived mainly from apoptotic and necrotic cells as part of normal cell turnover and tissue homeostasis. ctDNA serves as a noninvasive biomarker for detecting HNC through the detection of mutations and atypical fragment patterns [6]. Sarah et.al found that blood and saliva were found to be good sources of HPV-ctDNA. The presence of ctDNA strongly correlated with treatment response, demonstrating clinical utility as a non-invasive biomarker to monitor tumor progression in HPV-positive HNC [7]. A study demonstrated that pre-surgical saliva samples collected from patients with oral squamous cell carcinoma (OSCC) were utilized to detect the TP53 gene, whose mutations are in 73–100% of HPV-negative HNSCC cases, revealing the presence of the codon 72 polymorphism c.215C>G (p.Pro72Arg) in 10 patients (67%) and a heterozygous mutation at codon 172 c.514 G>T (p.Val172Phe) in 2 patients (13%) [8]. These findings suggest a potential for clinical applications of saliva detection for identifying genetic alterations in candidate biomarkers associated with oral cancer (OC) [9]. Leung et al. in 2021 demonstrated that elevated levels of cfDNA in saliva correlate with advanced staging of HNC, most notably in patients with HPV-positive squamous cell carcinoma [10]. Another investigation, which enrolled 11 patients and collected plasma and saliva after surgery at 1, 3, and 6 months, indicated that ctDNA was detected earlier using liquid biopsy compared to conventional monitoring techniques. Notably, patients without recurrence exhibited decreased ctDNA allele frequency post-treatment [11]. Wang et al. discovered 100% and 80% ctDNA in saliva and plasma samples of oral cavity cancers, respectively [12]. This implies that saliva, being more proximal to the head and neck, should serve as an earlier indicator of tumor development. Different techniques exhibit varying efficacy in collecting cfDNA. Some researchers assessed the ability to detect HPV DNA from patient saliva samples and found that the Oragene OG-600 receptacle yielded the highest concentration of total salivary DNA along with short fragments <300 bp corresponding to mononucleosomal cfDNA. This result underscores optimal techniques for isolating DNA from saliva, which will contribute to future applications in liquid biopsy-based cancer detection [13].

DNA methylation is a crucial epigenetic modification involving the addition of a methyl group to the cytosine ring within CpG dinucleotides, modifying gene expression without altering the underlying DNA sequence. Cancer-related DNA methylation changes often precede detectable gene mutations, serving as an early event in tumorigenesis. Across multiple studies, the EDNRB gene was shown to be silenced by hypermethylation in saliva samples of HNC patients [14]. Schussel et al. analyzed the saliva of 191 HNC patients and found *EDNRB* and *DCC* gene hypermethylation to be associated with HNC diagnosis with 75% sensitivity and 48% specificity [15]. RASSF1A, a tumor suppressor gene, has hypermethylation in the promoter region, leading to epigenetic inactivation. Ovchinnikov et al. analyzed RASSF1A in 143 patients with HNC and 46 controls, and this gene, alongside DAPK1, could discriminate patients in the early stages of HNC as compared to controls with 80% sensitivity and 87% specificity [16]. These genes, among others, show hypermethylation profiles that may serve as biomarkers for disease detection and monitoring. A study with a sample size of up to 987 has shown that a significant increase in EBV methylation was found in nasopharyngeal carcinoma (NPC) patients compared with controls. The methylated score of the EBV genome obtained by capture sequencing could distinguish NPC from controls [16]. This research study provided an appealing alternative for the non-invasive detection of NPC without a clinical setting. It paved the way for conducting a home-based large-scale screening in the future.

While notable, several biomarkers detected by hypermethylation (such as the *EDNRB* gene) offer only moderate specificity and sensitivity. These levels are too low for reliable clinical use. There is a need for more rigorous and accurate testing for enhanced diagnostic accuracy. Recently, genome-wide approaches to explore widespread DNA methylation, rather than more careful single-targeted gene assays, have been utilized to evaluate CpG loci and genes.

However, there are also some limitations in cfDNA detection from saliva. Firstly, saliva contains a significant amount of genomic DNA derived from oral epithelial cells, bacteria, and food particles, which can contaminate cfDNA and complicate the analysis. This contamination may obscure cfDNA signals or reduce the overall sensitivity of the test. Secondly, compared to blood-based cfDNA assays, standardized protocols for saliva cfDNA collection, stabilization, and analysis are less well-established, leading to variability in results. Despite these challenges, ongoing advancements in technology and a deeper understanding of cfDNA biology could enhance the utility of saliva-based cfDNA testing. Table 1 summarizes the findings of various studies conducted involving ctDNA and DNA methylation and their notable findings.

### 2.2. mRNA and Non-Coding RNAs (ncRNAs)

mRNA is a critical molecule in the central dogma of molecular biology, serving as the intermediary between the transcription of genetic information from DNA and the translation of that information into proteins by ribosomes [17]. A seminal study by Li et al. performed microarray analysis of saliva from OC patients, identifying a panel of overexpressed mRNA biomarkers, including OAZ1, DUSP1, S100P, and SAT1 [18]. This marker panel achieved 89% sensitivity and 78% specificity in distinguishing OC patients from healthy controls [19]. Subsequent validations by Elashoff et al. showed similar results, but the area under the curve (AUC) values were lower than previously reported [20].

ncRNAs are RNA molecules that are not translated into proteins but play crucial regulatory roles in various cellular processes. ncRNAs, particularly microRNAs (miRNAs) and long non-coding RNAs (lncRNAs), have emerged as significant players in the prediction, prognosis, and pathogenesis. ncRNAs are gaining attention as potential diagnostic and prognostic biomarkers and as therapeutic targets in HNC. In HNC, specific miRNA signatures have been identified that correlate with clinical outcomes and may provide a foundation for targeted therapies. A study indicated that the expression levels of salivary miR-21 and -155 in healthy volunteers were 2.49 and 2.84 times lower, respectively, than those in OSCC patients (*p* < 0.05). Positive associations were found between the expression levels of miR-21 and miR-155, while a negative correlation was observed for miR-375 concerning the T-index according to TNM classification (r = 0.68, r = 0.75, and r = −0.67, respectively), as well as with lymph node metastasis presence (r = 0.78, r = 0.71, and r = −0.59, respectively). Patients exhibiting a favorable response to neoadjuvant chemotherapy had lower levels of miR-21 and -155 but higher levels of miR-375 in saliva compared to those who were resistant [21]. Another study demonstrated that salivary levels of miR-200 and miR-34 were decreased in OSCC patients relative to healthy individuals; conversely, the expression level of miR-24 was increased in OSCC patients compared to healthy controls [22]. Wu et al. found that salivary miRNAs can be detected as potential noninvasive biomarkers for the detection of NPC, and differentially expressed miRNAs in saliva might play critical roles in NPC by regulating their target genes, which are associated with some significant pathways, such as the p53 signaling pathway [23]. Well-documented lncRNAs, such as MALAT1 and HOTAIR, are present in the saliva of OSCC patients; notably, patients with lymph node metastasis exhibit higher HOTAIR expression than those with non-metastatic patients [24]. Salivary LINC00657 may effectively differentiate OSCC from potentially malignant disorders with considerable diagnostic accuracy; additionally, low levels of salivary miR-106a could indicate malignancy potential [25]. The stability of circulating mRNA and ncRNA signatures has supported their promise as accessible noninvasive biomarkers. Table 2 summarizes the findings of various studies conducted involving mRNA and ncRNAs and their notable findings.

### 2.3. Circulating Tumor Cells (CTCs)

CTCs represent cancer cells entering the vasculature and spreading to distant sites [26]. Many different methods have been established to isolate CTCs, including the utilization of tumor cell-specific ligands. Integrins, cell surface receptors highly expressed in many tumor types, are one such target [27]. Cancers located in or near the oral cavity may directly shed cells into saliva. Therefore, saliva-derived CTCs represent a more promising application for detecting tumor cells in head and neck cancers. CTCs can predict recurrence and survival outcomes in patients with HNC. A study from Australia demonstrated that patients with detectable CTCs at follow-up appointments were 2.5 times more likely to experience recurrence or mortality from HNSCC before their next clinical visit [28]. In another study conducted by Jatana et al. in which CTCs were obtained in patients with HNSCC undergoing surgical intervention, patients with no detectable CTCs had a higher probability of disease-free survival. On the other hand, patients with >25 CTCs/mL were more likely to have worse clinical outcomes [29]. Similarly, studies evaluating the application of CTCs in monitoring treatment response are ongoing. For example, Wang et al. investigated CTC counts in 47 HNSCC patients undergoing concurrent chemoradiotherapy. CTC counts in this population correlated directly with progression-free survival and overall survival [30].

Furthermore, links between CTCs and immunotherapy have been noted in multiple studies. Programmed death 1 (PD-1) checkpoint inhibitors may block the PD-1/PD-L1 immune checkpoint pathway on CTCs and activate the immune system to eliminate CTCs present in the circulation. It has been proposed that patients with high baseline PD-L1 expression could benefit from adjuvant immunotherapy targeting the PD-1/PDL1 axis [31]. However, there are currently limited studies on CTCs in saliva; given the extensive research conducted on other bodily fluids, CTCs in saliva likely present a promising avenue for future investigation. Table 3 summarizes the findings of various studies conducted involving CTCs and their notable findings.

### 2.4. Cytokines

Alterations in the tumor microenvironment (TME) play a critical role in regulating the hallmarks of cancer development, including evasion of apoptosis, metastasis, and angiogenesis, among others. For example, a major cell type in the TME is cancer-associated fibroblasts (CAFs). CAFs secrete a wide variety of cytokines essential for inflammation, cell proliferation, and tumor growth [32]. Cytokines, small secretory proteins generated by neoplastic and stromal cells, orchestrate intricate and dynamic cell–cell interactions within the TME. They are key regulators of both inflammation and immune responses, exerting their effects via multiple signaling cascades, including the mitogen-activated protein kinase (MAPK), nuclear factor-kappa B (NF-kB), phosphoinositide 3-kinase (PI3K), and the Smad2/AKT pathways. In the context of cancer-related inflammation and immune evasion, cytokines play significant roles. In HNC, cytokines are upregulated in TME, promoting tumor progression and metastatic behavior. One study analyzed 1022 oral saliva samples, including 157 from OC patients and 865 from healthy controls, indicating that salivary CPT1A may serve as a predictive marker, thereby enhancing diagnostic accuracy for OC [33]. The study of Borislav et al. demonstrated that saliva is a valuable specimen for investigating cachexia and identified IL-13 and TGF-β as potential biomarkers for cancer cachexia [34]. A systematic review and meta-analysis of 23 studies conducted by Chiamulera et al. evaluated different pro-inflammatory and anti-inflammatory salivary cytokines as potential biomarkers for OC [35]. Levels of various salivary cytokines were analyzed for differences among healthy controls (HC), OC, and the potential malignant oral disorder group (PMOD). The results showed a significant increase in levels of IL-8, IL-6, TNF-α, IL1Β, and IL-10 in the OC group compared to the HC group. In addition, levels of IL-8, IL-6, TNF-α, and IL-1β were noted to be significantly higher in the OC group compared to the PMOD group. Of note, there was notable heterogeneity in the results of this study, attributed to variations in saliva collection and quantification methods. Multiple studies have demonstrated increased IL-8 expression in the setting of HNC. For example, during salivary biomarker investigation, IL-1B and IL-8 concentrations were elevated in OSCC patients compared to patients with oral dysplasia [36]. Another study also indicated that IL-6 is the most relevant cytokine for the development and severity of oral mucositis. Considering its role in the progression of OSCC, IL-6 can be regarded as an important early biomarker [37]. Cytokines contribute to tumor growth and metastasis in HNC. Therefore, closer exploration may aid in developing novel therapeutic agents targeting specific cytokines and receptors in the treatment of HNC. Further studies highlighting the contribution of individual pathways will assist in achieving better therapeutic outcomes in HNC. Table 4 summarizes the findings of various studies conducted involving cytokines and their notable findings.

### 2.5. Proteomics

The cellular and molecular heterogeneity of OC and the large number of genes potentially involved in oral carcinogenesis emphasize the importance of studying gene expression changes on a global scale by proteomics [38].

Proteins are essential molecules involved in pathological processes of OC growth, apoptosis, and metastasis. Proteins secreted in saliva, such as hormones, antibodies, and enzymes, can provide comprehensive information about OC and may be potential targets for noninvasive screening. Although concentrations of these proteins in saliva may be up to 15 times lower than in plasma, technological advancements have allowed for improved sensitivity and specificity of detection in saliva [39].

The analysis of salivary proteomes has been useful in identifying possible biomarkers for OSCC. In a study by Krapfenbauer et al., 25 proteins were linked to OSCC and raised interest as potential biomarkers for OC [40]. Out of these 25 proteins, 12 had never been reported in the past: protein galectin-7, cofilin, CRP precursor, creatine kinase, m-chain, fatty acid binding protein, keratin type II, myosin light chain 2 and 3, nucleoside diphosphate kinase A, phosphoglycerate mutase 1, plakoglobin, and retinoic acid binding protein II [34]. It has long been theorized that acute-phase response proteins (APPs) can be used as markers for OSCC due to inflammation present with malignancy. Among APPs, haptoglobin B (HAP-B), a-antitrypsin (AATa), complement-C3, hemopexin (HPX), serotransferrin, transthyretin (TTR), and fibrinogen B (FIB-B) have all been detected in the saliva of patients with OSCC and can be used as potential biomarkers for the early detection of OSCC [41]. Continued advances and discoveries into protein biomarkers are ongoing. Resistin (RETN) is a cysteine-rich adipose-derived peptide hormone involved in inflammatory processes that have shown a correlation with late-stage OSCC and lymph node metastasis [42]. Promising proteins, such as alpha-amylase (HPA), human salivary amylase (sAA), keratin-10 (K-10), and human serum albumin (GA-HSA), have all been implicated as salivary biomarkers for OSCC [43].

Different analytical techniques have been utilized to analyze salivary proteins. Two-dimensional gel electrophoresis (2DGE) is the most basic technology for separating complex protein mixtures. Shortcomings of 2DGE include highly abundant proteins obscuring less abundant proteins in mixtures and small proteins with acidic or basic isoelectric points migrating outside of analysis ranges [44]. As a result, technologies such as mass spectrometry (MS) and matrix-assisted laser desorption ionization (MALDI) have been used instead. MS, with high speed and high sensitivity, allows the examination of salivary proteomes at the level of gene expression and posttranslational modifications [45]. While powerful, these technologies are costly and may not be routinely accessible in clinical practice. This hinders their use in day-to-day practice. Attempts have been made to overcome the challenges faced by proteomic analysis. These include longer fasting periods prior to the collection of saliva for diagnosis to improve the discriminatory capacity of any method of analysis. Results from the study using this technique demonstrated that five markers, Mac-2 binding protein (M2BP), migration inhibitory factor-related protein 14 (MRP14), CD59 glycoprotein, catalase, and profilin provide 90% sensitivity and 83% specificity for OSCC detection [46]. Lin developed a new method for evaluating the capability of saliva analysis combining membrane protein purification with surface-enhanced Raman spectroscopy (SERS) to analyze and classify the saliva protein SERS spectra from NPC and healthy subjects. Diagnostic sensitivity of 70.7%, specificity of 70.3%, and diagnostic accuracy of 70.5% could be achieved by PCA-LDA for NPC identification [47].

Salivary proteins have been the subject of study due to the numerous proteins present in saliva, many of which have been linked to inflammation and lymph node metastasis in prior studies. Notable challenges in the utilization of salivary proteomic biomarkers include significant variability present in identification and environmental factors, such as proteolytic enzymes and oral microorganisms affecting the samples collected [4]. Table 5 summarizes the findings of various studies conducted involving proteomics and their notable findings.

### 2.6. Metabolomics

Metabolomics is the field of study focused on the identification of metabolites produced during the metabolism of body fluids, cells, and tissues. Elevated levels of phenylalanine and tyrosine have been reported in patients with OC [48]. Additionally, organic acids such as lactate, acetate, and citrate are often involved in the altered energy metabolism observed in cancer cells—a phenomenon known as the Warburg effect—whereby cancer cells rely on glycolysis even under oxygen-rich conditions. Lactate dehydrogenase (LDH) is an enzyme that plays a crucial role in this process by facilitating anaerobic metabolism. Increased levels of LDH also serve as a prognostic marker for head and neck cancer (HNC). A meta-analysis involving 642 HNSCC patients revealed that salivary LDH levels were significantly higher in the HNSCC group compared to controls, suggesting that LDH can be utilized as a valuable minimally invasive biomarker for screening and prevention of head and neck cancers [49]. Similarly, salivary short-chain fatty acids (SCFAs) could serve as indicators for the early diagnosis of systemic diseases including HNC [50].

Certain salivary metabolites, including d-glycerate-2-phosphate, pseudouridine, 1-methylhistidine, 2-oxoarginine, and sphinganine-1-phosphate, among others, have been identified to try to differentiate between malignant and precancerous lesions [51]. Sugimoto et al. analyzed profiles in OC patients. They identified glutamic acid, choline, threonine, beta-alanine, tryptophan, and leucine, among others, as consistently elevated in saliva and tumor tissues of patient samples as compared to controls [52]. The glucose-alanine cycle, the urea cycle, and other metabolic pathways (such as gluconeogenesis) are altered in HNC, which may explain the elevations in specific metabolic markers in the setting of malignancy [53]. While multiple studies have utilized salivary metabolomics to detect HNC, inconsistencies in the metabolite profiles have thus far limited the clinical ability of this approach. Table 6 summarizes the findings of various studies conducted involving metabolomics and their notable findings.

### 2.7. Exosomes

Exosomes are extracellular vesicles that exist stably in various body fluids, including saliva and blood. They are surrounded by a phospholipid bilayer and there is heterogeneity in the substances present, including proteins, RNA, DNA, and lipids [54]. Tumor-derived exosomes are abundant in the plasma of HNSCC patients [55]. Saliva-derived exosomes from HNSCC patients were shown to carry high amounts of CD44v3, PDL1, and CD39, per a study in which exosomes were isolated from HNSCC patients via ultracentrifugation [56]. Furthermore, HNSCC patients’ exosomes were found to be potent producers of immunosuppressive adenosine [56].

Cancer cells can selectively package specific miRNAs into exosomes, and these selective miRNAs can act as tumor suppressors in target cells. Alterations in miRNA expression or processing also occur in HNC development. Faur et al. reviewed salivary exosomal miRNAs exhibiting altered expression correlating with OC progression, highlighting exosomal miR-21 and miR-451 as the most consistently changed [57]. Langevin et al. isolated exosomes from patients with HNC and compared levels of miRNAs present in exosomes. The results showed that miRNA expression in HNC cell exosomes significantly differed from normal oral epithelial cells. Certain exosomal miRNAs (miR-486-5p, miR-486-3p, miR-10b-5p) were noted to be secreted by cancer cells. In this study, miR-10b-5p displayed a specificity of 100% but a low sensitivity of 18% in the detection of cancer [58]. Among the miRNA studied, miR-486-5p was able to detect stage I cancer [58]. A separate study comparing miRNA in the plasma of patients with HNC before and after treatment showed that the expression of specific miRNAs, namely miR-142-3p, miR-186-5p, miR-195-5p, miR-374b-5p, and miR-574-3p, was associated with a poorer prognosis [58].

Of note, certain miRNAs, such as miR-200a, have been linked with smoking-induced epigenetic changes [57]. In patients with non-cancerous conditions, including smoking and autoimmune diseases causing significant inflammation, there may be miRNA expression alteration that could interfere with results and the cancer diagnosis process [59]. For example, miR-24-3p is expressed at different levels in the elderly compared to young adults [60]. Factors such as age, tobacco use, and inflammation-altering biomarker levels complicate their use for cancer diagnosis by reducing diagnostic accuracy. Table 7 summarizes the findings of various studies conducted involving exosomes and their notable findings.

## 3. Emerging Technologies in Saliva Detection

Recent advancements have brought forth a variety of innovative technologies for detecting and analyzing components in saliva. This article explores the latest trends and breakthroughs in saliva-based testing and examines their potential to shape the future of diagnostics.

### 3.1. Next-Generation Sequencing (NGS) and Digital Droplet PCR

NGS, with its high throughput and deep sequencing capabilities, is an ideal method for saliva testing. As NGS technology rapidly advances, saliva-based testing has become highly refined and reliable. Recent advancements have markedly improved its sensitivity in identifying low abundance ctDNA found in saliva. A significant study demonstrated that NGS can effectively identify fusions such as ETV6-NTRK3, MYB-NFIB, and EWSR1-ATF1 from saliva specimens [61]. Nevertheless, challenges remain; saliva consists of a heterogeneous mixture of DNA from various sources, complicating both the extraction process and precise targeting of tumor-specific genetic material. High-quality analyses require advanced techniques to accurately differentiate and quantify tumor signals. Furthermore, saliva may contain microbial DNA, contaminants, or non-cancerous cell-derived DNA that could potentially obscure analytical results.

Digital Droplet PCR (ddPCR) is another highly sensitive and precise method for quantifying DNA or RNA in saliva detection. It is particularly useful in detecting rare genetic mutations or low concentrations of target molecules. A meta-analysis extracted from 33 studies showed that ddPCR-based testing exhibited the highest diagnostics odds ratio at 138 (59.5, 318), followed closely by next-generation sequencing (NGS) at 120 (39.7, 362) [62]. This showed that ddPCR is also applicable to the detection of saliva components and has a lower cost.

### 3.2. Mass Spectrometry (MS)

MS is a powerful analytical technique used to identify and quantify proteins and metabolites in biological samples, including saliva. MS can simultaneously analyze a wide range of proteins and metabolites, providing a comprehensive profile of the biochemical and molecular changes associated with cancer. In one study, Michał identified peptides derived from proteins previously suggested as potential biomarkers for salivary gland tumors, such as ANXA1, BPIFA2, FGB, GAPDH, HSPB1, IGHG1, and VIM, as well as for tumors in other tissues or organs, including SERPINA1, APOA2, CSTB, GSTP1, S100A8, S100A9, and TPI1 [63]. Unfortunately, none of these identified peptides were present in all analyzed samples. Another study demonstrated the potential of PIK3CA hotspot mutations in saliva as a diagnostic marker for OSCC patients using MS [64]. Research identifying salivary autoantibodies as OSCC biomarkers suggested that proteins such as LMAN2, PTGR1, RAB13, and UQCRC2 could be further developed to improve early diagnosis of OSCC [65]. Additionally, gas chromatography coupled with mass spectrometry (GC-MS) identified metabolites like malic acid, maltose, protocatechuic acid, lactose, 2-ketoadipic acid, and catechol as differentially expressed in OSCC compared to control individuals from South America [66].

### 3.3. Other New Technologies

Nanotechnology is transforming saliva diagnostics by allowing the detection of biomarkers at very low concentrations. With the use of nanoparticles and biosensors, it is possible to identify specific disease markers in saliva that were previously impossible to detect. Smart and intelligent designs of the biosensors offer promising prospects for portable, flexible, multifunctional, and efficient operation, which enable the real-time and fast in vivo detection of interest biomarkers in saliva. Jafari et al. built an immunosensor by immobilizing anti-Cyfra21.1 on a cysteamine (CysA) and glutaraldehyde (GA)-modified gold electrode. The platform provides a low-cost, reliable, and robust method to perform non-invasive detection of salivary Cyfra21.1 [67]. Li et al. developed a method using opal photonic crystals (OPC) to enhance upconversion fluorescence for the detection of carcinoembryonic antigen (CEA) in saliva [68]. The use of upconversion nanoparticles increases the biosensor’s sensitivity, making it an effective tool for early detection and monitoring that patients can perform at home [69,70].

## 4. Perspectives and Challenges

Identifying specific molecular biomarkers for diagnosis is a promising avenue for overcoming limitations presented by traditional invasive diagnostic methods. Liquid biopsy-based detection would offer a less-invasive safer alternative method for early disease diagnosis and prognosis. Among the options for liquid biopsy, saliva has advantages due to decreased cost, lack of discomfort, and ease of collection [3]. Saliva can also reflect biological changes relevant to disease, as it contains various biomolecules, including DNA, RNA, proteins, and metabolites, which can serve as biomarkers for cancer detection and the prediction of cancer treatment outcomes (Figure 1). Table 8 summarizes prior studies that have reviewed the literature in recent years.

Despite this promise, many challenges stand in the way of the widespread adoption of these exciting avenues. The composition of saliva can vary due to factors such as circadian rhythms, diet, oral health, and hydration levels, potentially affecting the reliability of test results. Future studies will need to further develop these associations and how they interfere with results. Currently, there is a lack of standardization in saliva quantification. This has led to inconsistent results across multiple studies, making it difficult to establish uniform diagnostic criteria. The inconsistencies seen between different studies highlight the need for larger-scale validation and reproducibility testing. Adequately powered validation studies using standardized methods will be critical to translating altered salivary patterns into viable clinical tests for early HNC detection and prognostication. Establishing standardized protocols for saliva collection and analysis can help reduce the level of variation currently seen. Many studies in the current literature have relatively small sample sizes, with only moderate specificity and sensitivity seen across multiple markers. Larger multi-center studies and longitudinal studies are required to validate the findings seen thus far and further establish biomarker changes over time to establish their clinical utility. Meanwhile, saliva typically contains biomarkers at lower concentrations compared to blood, which can limit the sensitivity and specificity of saliva-based tests. More precise technologies need to be developed to detect trace amounts of material.

## 5. Conclusions

Saliva-based biomarkers represent a transformative approach in the field of HNC diagnostics and treatment, offering a noninvasive and cost-effective means to detect and monitor cancer progression. Advances in genomic, transcriptomic, metabolomic, and proteomic profiling of saliva pave the way for more precise, early, and personalized management of HNC patients. Continued research efforts and clinical validation studies are essential to unlock the full potential of saliva-based biomarkers in improving patient outcomes and reducing the mortality of HNC worldwide.

## Figures and Tables

**Table 1:** Application of saliva-based cfDNA/ctDNA and DNA methylation.

Title of Study	Cases/Controls	Type of Change	Application	Findings
Blood and saliva-derived ctDNA is a marker of residual disease after treatment and correlates with recurrence in human papillomavirus-associated head and neck cancer (2023) [7]	235 blood and saliva samples were collected from 60 HPV-positive (cases) and 17 HPV-negative (controls) before and after treatment	ctDNA levels, before and after treatment	Prognosis, Disease Response and Progression	HPV-ctDNA detection was significantly higher prior to treatment (91%) than after treatment; all patients positive for ctDNA before treatment showed significant reductions in ctDNA levels post-treatment
Non-Invasive Saliva-based Detection of Gene Mutations in Oral Cancer Patients by Oral Rub and Rinse Technique (2021) [8]	Tissue samples of 15 patients with oral cancer were obtained orally (case) and during the surgical excision of the cancerous oral lesion (control)	Genomic DNA, TP53 gene sequencing from tissue DNA	Diagnosis	Oral rinse and rub technique matched salivary DNA results for 6 samples; 4 samples displayed the same genetic changes at codon 72.
Detection of somatic mutations and HPV in the saliva and plasma of patients with HNSCC (2015) [12]	93 patients with HNSCC and 3 with OSCC	ctDNA mutations	Diagnosis and prognosis	Evaluation of ctDNA in patients with HNSCC, salivary ctDNA mutations carried 100% specificity for cancer detection.

**Table 2:** Application of saliva-based mRNA and ncRNAs.

Title of Study	Cases/Controls	Type of Change	Application	Findings
Oral Squamous Cell Carcinoma Detection By Salivary Biomarkers in a Serbian Population (2011) [19]	35 patients with recently diagnosed and untreated OSCC in a Serbian population (cases) and 51 patients without OSCC (controls)	Salivary protein markers and salivary mRNA markers	Diagnosis	4 out of 6 salivary markers (IL-8, IL-1B, SAT-1, and S100P) were elevated in OSCC patients and could discriminate between cancer and control subjects.
Pre-Validation of Salivary Biomarkers for Oral Cancer Detection (2012) [20]	395 subjects from 5 independent cohorts in case (OSCC) vs. control	Expression of 7 mRNA and 3 protein markers	Diagnosis	Expression of all 7 mRNA and 3 protein markers was increased in OSCC in all cohorts; IL-8 and SAT, of the individual makers), had statistical significance.
Clinical Significance of Salivary MIR-21, -155, and -375 in Patients with Squamous Cell Carcinoma of Oral Cavity (2024) [21]	61 patients with Stage II-IV OSCC analyzed for expression levels of miR-21, -155, and -375 as compared to controls without disease	mRNA expression levels	Diagnosis and prognosis	miR-21 and -155 expression levels in healthy volunteers were lower than in OSCC patients; patients with response to neoadjuvant chemotherapy had lower miR-21 and -155 levels and higher miR-375 levels in saliva as compared to those with resistant tumors
Biomarkers for Oral Squamous Cell Carcinoma (miR-24, miR-200, and miR-34): Screening and Detection MicroRNA (2024) [22]	30 patients with OSCC and 30 healthy individuals analyzed for miR-24, miR-200, and miR-34 in saliva	mRNA expression levels	Diagnosis	miR-200 and miR-34 expression levels were decreased in OSCC patients and as compared to controls; and miR-24 expression levels was increased in patients with OSCC
Genome-wide study of salivary microRNAs as potential noninvasive biomarkers for detection of nasopharyngeal carcinoma (2019) [23]	22 patients with newly diagnosed nasopharyngeal cancer and 25 healthy controls had miRNA expression profiling completed	mRNA expression levels	Diagnosis	The twelve dysregulated miRNAs screened separated nasopharyngeal cancer patients from healthy controls with high sensitivity and specificity
Salivary LINC00657 and miRNA-106a as diagnostic biomarkers for oral squamous cell carcinoma, an observational diagnostic study (2023) [25]	36 total participants were included, of which 12 patients had diagnosed OSCC; 12 patients had oral lichen planus and 12 individuals had no disease	mRNA expression levels	Diagnosis	OSCC patients showed the highest LINC00657 and lowest miR-106a fold change among all groups; LINC00657 was able to differentiated OSCC grades II and III with diagnostic accuracy of 83.3%

**Table 3:** Application of circulating tumor cells.

Title of Study	Cases/Controls	Type of Change	Application	Findings
Circulating tumour cells predict recurrences and survival in head and neck squamous cell carcinoma patients (2024) [28]	154 HNSCC patients were followed for 4.5 years and samples were collected at baseline, 3 months, 6 months, 1 year and 2 years post-treatment	Expression levels of CTCs	Prognosis, Surveillance	In HNSCC patients who died from cancer, each patient (100%) had at least 1 CTC in their baseline sample, which was more than patients who survived during the follow up period (45.04%); patients who died had a mean of 2.19 CTCs as compared to 1.64 in patients who survived (*p* < 0.01).
Significance of circulating tumor cells in patients with squamous cell carcinoma of the head and neck: initial results (2010) [29]	48 patients with SCCHN who underwent surgical intervention were followed for a mean of 19.0 months	Expression level of CTCs	Prognosis	Patients with no detectable CTCs/mL of blood had a significantly higher probability of disease-free survival (*p* = 0.01)
Prognostic significance of PD-L1 expression on circulating tumor cells in patients with head and neck squamous cell carcinoma (2017) [31]	113 patients with locally advanced HNSCC had PD-L1 expression evaluated at baseline, after 2 cycles of chemotherapy, and at the end of concurrent chemoradiotherapy	PD-L1 expression level on CTCs	Prognosis	Patients with CTCs overexpressing PD-L1 at the end of treatment had shorter progression-free survival and overall survival (*p* < 0.01).

**Table 4:** Application of saliva-based cytokines.

Title of Study	Cases/Controls	Type of Change	Application	Findings
Oral Microbiome and CPT1A Function in Fatty Acid Metabolism in Oral Cancer (2024) [33]	1022 oral saliva samples were analyzed, 157 from OSCC patients and 865 from healthy controls to identify fatty acids and cytokines	Levels of bacteria expression in the oral microbiome, fatty acids and cytokines expression	Diagnosis	Patients with oral cancer had a higher abundance of Streptococcus and Parvimonas expression, which correlated positively with increased IL-6 and TNF-a expression. This was linked to a poorer disease-free survival and overall survival.
Salivary Biomarkers for Detection of Oral Squamous Cell Carcinoma in a Taiwanese Population (2016) [36]	180 samples were collected, 60 from OSCC patients, 60 from controls, and 60 from patients with potential malignancy	Cytokine and proteomic expression	Diagnosis	Protein concentrations of Il8 and IL1B were significantly higher in OSCC patients than controls and dysplasia patients

**Table 5:** Application of saliva-based proteomics.

Title of Study	Cases/Controls	Type of Change	Application	Findings
Identification of tumour-related proteins as potential screening markers by proteome analysis-protein profiles of human saliva as a predictive and prognostic tool (2014) [40]	Saliva from 5 patients without disease and 5 patients with suspicion for OSCC were collected	Protein Expression	Diagnosis	25 proteins were identified in the saliva of all patients with suspicion for OSCC, but not in healthy individuals
Aberrant proteins in the saliva of patients with oral squamous cell carcinoma (2013) [41]	Saliva of 12 patients with diagnosed OSCC (cases) and 12 patients without disease (controls) were utilized to identify protein biomarkers	Protein Expression	Diagnosis	Hemopexin and a-1B glycoprotein were detected in patients with OSCC but not in controls; Significantly altered levels of AAT, C3, transferrin, transthyretin, and HAP were detected in patients with OSCC
Saliva proteome profiling reveals potential salivary biomarkers for detection of oral cavity squamous cell carcinoma (2015) [42]	Saliva proteomes were assessed between patients with no disease, individuals with oral potentially malignant disorders, and OSCC patients	Protein Expression	Prognosis	22 overexpressed salivary proteins were identified in the OSCC group as compared to the healthy controls and patients with suspicion for malignancy; RETN levels in OSCC patients were significantly higher than in the other groups

**Table 6:** Application of saliva-based metabolomics.

Title of Study	Cases/Controls	Type of Change	Application	Findings
Oral Microbiome and CPT1A Function in Fatty Acid Metabolism in Oral Cancer (2024) [33]	1022 oral saliva samples were analyzed, 157 from OSCC patients and 865 from healthy controls to identify fatty acids and cytokines	Levels of bacteria expression in the oral microbiome, fatty acids, and cytokines expression	Diagnosis	Healthy controls exhibited increased levels of short-chain fatty acids (metabolite) as compared to patients with OSCC
Capillary electrophoresis mass spectrometry-based saliva metabolomics identified oral, breast and pancreatic cancer-specific profiles (2010) [52]	215 individuals, of which 69 had OSCC and 87 were healthy controls were analyzed.	Comprehensive metabolite analysis	Diagnosis	57 principal metabolites were identified to accurately predict the probability of being affected by multiple specific cancers

**Table 7:** Application of saliva-based exosomes.

Title of Study	Cases/Controls	Type of Change	Application	Findings
Cargo and Functional Profile of Saliva-Derived Exosomes Reveals Biomarkers Specific for Head and Neck Cancer (2022) [56]	Exosomes were collected from the saliva of 21 HNSCC patients and 12 healthy donors.	Expression of immune checkpoints and tumor associated antigens on saliva-derived exosomes	Diagnosis	CD-63 captured exosomes of HNSCC patients carried high amounts of CD44v3, PDL1, and CD39 compared to controls.
Comprehensive microRNA-sequencing of exosomes derived from head and neck carcinoma cells in vitro reveals common secretion profiles and potential utility as salivary biomarkers (2017) [58]	4 discrete HNSCC cell clines from 4 different sites (buccal, pharynx, hypopharynx, and tongue) were compared to primary human gingival epithelial cells from 3 healthy female donors as comparison	Expression of miRNA transcripts from exosomes	Diagnosis	22 miRNA transcripts were detected in exosomes from at least 1 HNSCC cell line but not by control oral epithelial cells; 8 miRNA transcripts were secreted by all 4 HNSCC cell lines. miR-486-5p was detected in salivary exosomes from all samples, with more notable elevations in cases of HNSCC with a corresponding sensitivity of 45% and specificity of 89%; it was able to identify the lone early stage 1 case.

**Table 8:** Select studies of saliva as a liquid biopsy.

Study	Year of Publication	Liquid Biopsy Targets Discussed
Cabezas-Camarero, et al. [71]	2022	cfDNA, CTCs, extracellular vesicles, miRNA
Cristaldi, et al. [72]	2019	cfDNA, CTCs, extracellular vesicles, mRNA
Kong, et al. [27]	2021	cfDNA, CTCs, exosomes, metabolites, mRNA
Kumar, et al. [3]	2023	cfDNA, cytokines, DNA methylation, exosomes, miRNA
Patel, et al. [4]	2022	cfDNA, CTCs, ctNA, metabolites, vesicles

## Data Availability

Not applicable.
